# Collagen 24 α1 Is Increased in Insulin-Resistant Skeletal Muscle and Adipose Tissue

**DOI:** 10.3390/ijms21165738

**Published:** 2020-08-10

**Authors:** Xiong Weng, De Lin, Jeffrey T. J. Huang, Roland H. Stimson, David H. Wasserman, Li Kang

**Affiliations:** 1 Division of Systems Medicine, School of Medicine, University of Dundee, Dundee, Scotland DD1 9SY, UK; x.weng@dundee.ac.uk (X.W.); J.T.J.Huang@dundee.ac.uk (J.T.J.H.); 2 Drug Discovery Unit, University of Dundee, Dundee, Scotland DD1 9SY, UK; d.lin@dundee.ac.uk; 3 Centre for Cardiovascular Science, University of Edinburgh, Edinburgh, Scotland EH16 4TJ, UK; roland.stimson@ed.ac.uk; 4 Department of Molecular Physiology and Biophysics and Mouse Metabolic Phenotyping Centre, Vanderbilt University, Nashville, TN 37232, USA; david.wasserman@vanderbilt.edu

**Keywords:** extracellular matrix, collagen, obesity, diabetes

## Abstract

Aberrant extracellular matrix (ECM) remodelling in muscle, liver and adipose tissue is a key characteristic of obesity and insulin resistance. Despite its emerging importance, the effective ECM targets remain largely undefined due to limitations of current approaches. Here, we developed a novel ECM-specific mass spectrometry-based proteomics technique to characterise the global view of the ECM changes in the skeletal muscle and liver of mice after high fat (HF) diet feeding. We identified distinct signatures of HF-induced protein changes between skeletal muscle and liver where the ECM remodelling was more prominent in the muscle than liver. In particular, most muscle collagen isoforms were increased by HF diet feeding whereas the liver collagens were differentially but moderately affected highlighting a different role of the ECM remodelling in different tissues of obesity. Moreover, we identified a novel association between collagen 24α1 and insulin resistance in the skeletal muscle. Using quantitative gene expression analysis, we extended this association to the white adipose tissue. Importantly, *collagen 24α1* mRNA was increased in the visceral adipose tissue, but not the subcutaneous adipose tissue of obese diabetic subjects compared to lean controls, implying a potential pathogenic role of collagen 24α1 in obesity and type 2 diabetes.

## 1. Introduction

Insulin resistance is a hallmark of many metabolic conditions including obesity, Type 2 diabetes, cardiovascular disease and cancers, therefore representing a potential therapeutic target. A growing body of evidence suggests a link between the extracellular matrix (ECM) remodelling and insulin resistance [1,2]. The ECM is composed of the interstitial matrix (e.g., fibrillar collagens, fibronectin, hyaluronan) and the basement membrane (e.g., collagen IV and laminin), which provide the structural support for tissues and are required for cell adhesion and migration during growth, differentiation, morphogenesis and wound healing [3]. Increased deposition of various ECM components (e.g., collagens, hyaluronan and osteopontin) and their binding to the ECM receptors (e.g., integrins and CD44) have been linked to the development of obesity-associated insulin resistance in skeletal muscle [4,5,6], adipose tissue [7,8] and the liver [9].

Despite the emerging importance of ECM remodelling in insulin resistance, the effective ECM targets remain largely undefined. Thus, studies identifying targets in a large scale is immediately necessary. Several groups have used approaches that give a more global view of changes such as oligonucleotide microarray analysis and two-dimensional gel electrophoresis based tandem mass spectrometry analysis (2-DE/MS-MS) [10,11]. These global analyses have yielded a view of patterns of changes in pathways and generated new hypotheses. However, changes in mRNA levels generated from the transcriptomics analysis may not be mirrored by changes in protein abundance [12]. The 2-DE/MS-MS analysis revealed only a few differences in protein abundance and none of those were ECM proteins [10]. These challenges are addressed in the current study through the use of an initial decellularisation technique [13]. The decellularisation process greatly removes the cellular components of the tissue without disrupting the tissue architecture, therefore enriching the ECM proteins and allowing a comparative proteomic analysis.

Collagens are the most abundant components of the ECM, consisting of three α-helical chains made of the repetitious amino acid sequence glycine-X-Y, where X and Y are frequently proline or hydroxyproline. It was previously shown that collagen isoforms I (1), III (3), IV (4), V (5), VI (6) and XVIII (18) were increased in insulin-resistant tissues [1,14]. Collagen XXIV (24) was discovered as a new member of the fibril-forming family of collagen molecules and has been demonstrated to be crucial for osteoblastic differentiation and mineralisation [15,16]. Collagen 24 has not been previously studied in the context of metabolic regulation or insulin resistance. In the current study, we applied an ECM-specific mass spectrometry-based proteomics in decellularised tissues to characterise the complete view of ECM collagen protein changes associated with high fat (HF) diet-induced obesity and insulin resistance in both skeletal muscle and liver. We found dramatic differences in collagen signature in muscle and liver and identified collagen 24 α1 as a new collagen isoform, which was associated with skeletal muscle insulin resistance.

## 2. Results 

### 2.1. In Vivo Quantitative Proteomics of the ECM Compartment

HF diet (60% fat) feeding in mice for 20 weeks causes obesity, insulin resistance and hepatic steatosis [9]. The fasting blood glucose was also elevated in HF-fed mice (11.14 ± 0.36 mM, *n* = 36) when compared to those in chow-fed control mice (8.55 ± 0.51 mM, *n =*11) (*p* < 0.001). To investigate tissue-specific ECM remodelling in obese mice, gastrocnemius muscle and liver samples were analysed from mice fed a HF or chow control diet for 20 weeks. The ECM-specific mass spectrometry-based proteomics detected 78 proteins in muscle, of which 39 (50%) proteins were ECM proteins or contained ECM regions (full list in Supplemental Table S1). In contrast, 265 proteins were detected in the liver, of which 181 (68%) proteins were ECM proteins or contained ECM regions (full list in Supplemental Table S2). These data reveal that the initial decellularisation procedure successfully removed the majority of abundant cytosolic proteins to improve detection of the lower abundance ECM proteins (in non-cellularised liver tissues: 92% were cellular proteins; 8% cytoskeletal proteins; 0% ECM proteins). To test the initial hypothesis that the ECM-related pathways were increased in muscle and liver of HF-fed obese mice, we performed gene-annotation enrichment analysis and KEGG (Kyoto Encyclopaedia of Genes and Genomes) pathway mapping using the proteins with a ratio of HF/Chow >2-fold. Our results showed that in skeletal muscle, differentially increased proteins were mainly collagen isoform clusters, which were highly involved in pathways including protein digestion and absorption, ECM–receptor interaction, amoebiasis, platelet activation, focal adhesion and PI3K (Phosphoinositide 3-kinase)-Akt (Protein kinase B) signalling pathway (Table 1). In contrast, in the liver 2-fold increased proteins were mainly involved in metabolic pathways such as glycolysis and gluconeogenesis, metabolism of butanoate, fructose, mannose, pyruvate, carbon, fatty acids and amino acids (Table 1). In contrast to skeletal muscle, none of the collagen isoforms were mapped to the enriched pathways in liver. These results highlight a dramatic difference in protein and pathway changes between muscle and liver, where the ECM remodelling was more prominent in the muscle but not in the liver.

**Figure 1:**
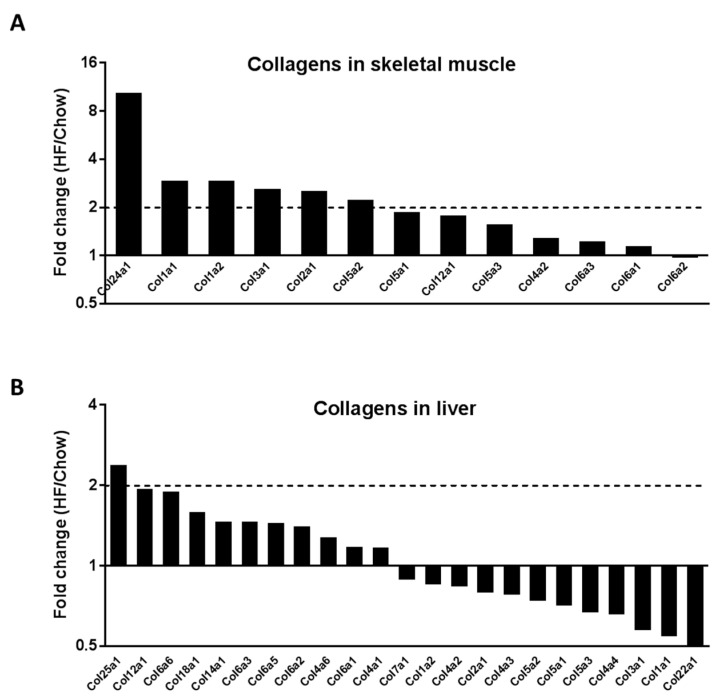
Proteomic detection of collagens in the extracellular matrix (ECM) of skeletal muscle and liver of chow- and high fat (HF)-fed mice. Gastrocnemius muscle and liver from chow- and HF-fed mice were decellularised by 1% SDS for 5–7 days before subjected to quantitative proteomics using iTRAQ (isobaric tags for relative and absolute quantitation) labelling peptide analysis. Data were presented as the ratio of the mean of samples from HF-fed mice over the mean of samples from chow-fed mice. (**A**) Collagen protein changes in the gastrocnemius muscle. *n* = 3:3. (**B**) Collagen protein changes in the liver. *n* = 3:4 (Chow:HF).

Collagens are the most abundant structural components of the ECM. We further examined the changes of collagen isoforms. The protein abundance ratio of HF/Chow was >1 in 12 out of the 13 collagen isoforms detected in the skeletal muscle, while Col24α1, Col1α1, Col1α2, Col3α1, Col2α1 and Col5α2 were increased by >2-fold in the HF-fed mice relative to chow-fed control mice (Figure 1A). In contrast, in the liver out of the 23 collagen isoforms detected, 11 collagens were shown with a ratio of HF/Chow >1 (Figure 1B). Moreover, the changes were moderate in liver with exception of a 2.36-fold increase in Col25α1 in the HF-fed mice relative to chow-fed control mice. These results suggest a very different signature of collagen changes between insulin-resistant muscle and liver. Unlike in the skeletal muscle, ECM collagens were not consistently increased by HF diet in liver.

**Figure 2:**
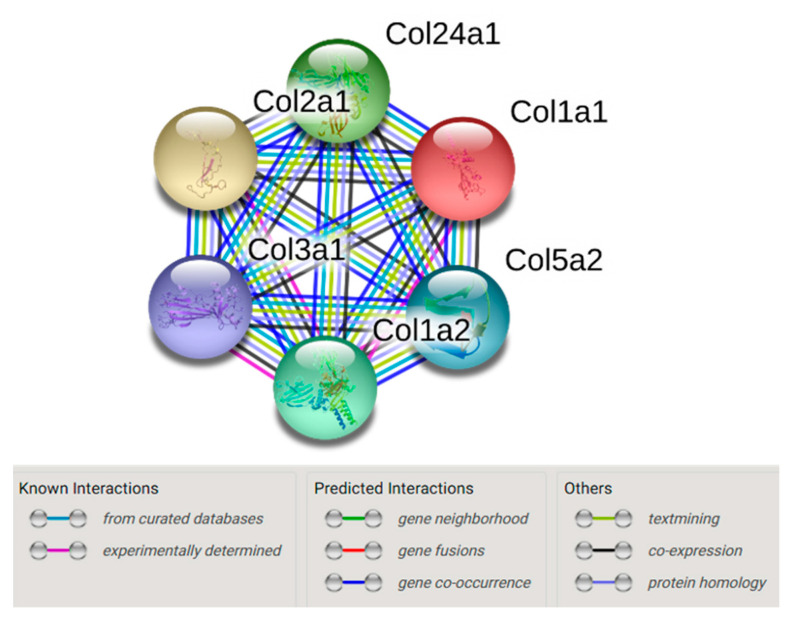
String network of the collagens that were increased by >2-fold in the muscle of HF-fed mice. Data was analysed using a mouse protein–protein interaction database (STRING, www.string-db.org) [17]. Coloured lines represent known or predicted interactions, or other associations as indicated.

As Col24α1 was increased by >10-fold in the muscle of insulin-resistant mice, we further examined its association with diet-induced insulin resistance. Collagen 24α1 is a fibril-forming collagen and it was not previously known to be associated with insulin resistance. Using a mouse protein–protein interaction database (STRING, www.string-db.org) [17], we showed that collagen 24α1 interacts with Col1α1, Col1α2, Col2α1, Col3α1 and Col5α2 (Figure 2), therefore providing evidence for how collagen 24α1 may be associated with insulin resistance.

### 2.2. Col24α1 mRNA Was Increased in Skeletal Muscle of HF-Fed Mice

**Figure 3:**
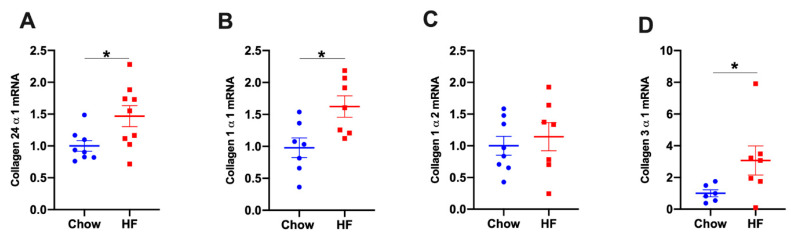
The mRNA levels of *Col24α1, Col1α1, Col1α2, and Col3α1* in gastrocnemius muscle of chow- and high fat (HF)-fed mice. (**A**) *Col24α1* mRNA expression, *n* = 8:9 (Chow:HF); (**B**) *Col1α1* mRNA expression, *n* = 7:7; (**C**) *Col1α2* mRNA expression, *n* = 8:7; (**D**) *Col3α1* mRNA expression, *n* = 6:7. Relative mRNA expressions were normalised to *GAPDH.* Data were presented as means ± SEM, * *p* < 0.05. HF: high fat.

To investigate the mechanism by which collagens were increased in the skeletal muscle of HF-fed mice, we measured the gene expression of various collagen isoforms. In gastrocnemius muscle, mRNA levels of *Col24α1*, *Col1α1* and *Col3α1* (but not *Col1α2*) were increased in HF-fed mice relative to chow-fed mice, suggesting increased Col24*α*1, Col1*α*1 and Col3*α*1 proteins were due to increased transcripts (Figure 3).

### 2.3. Col24α1 mRNA Was Increased in White Adipose Tissue, but Not in Liver of HF-Fed Mice

**Figure 4:**
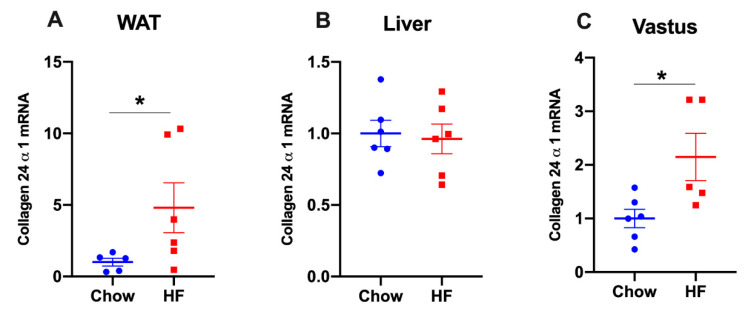
The mRNA levels of *Col24α1* in other metabolic tissues of chow- and high fat (HF)-fed mice. *Col24α1* mRNA expression was measured in epididymal adipose tissue ((**A**), *n* = 5:6), liver ((**B**), *n* = 6:6), and the superficial vastus lateralis ((**C**), *n* = 6:5). Relative mRNA expression was normalised to *18s* in epididymal adipose tissue and *GAPDH* in liver and vastus. Data were presented as means ± SEM, * = *p* < 0.05. HF: high fat. WAT: white adipose tissue.

We further examined whether the *Col24α1* gene was correspondingly increased in other metabolic tissues of HF-fed mice and found that *Col24α1* mRNAs were increased in epididymal white adipose tissue (WAT) and superficial vastus lateralis, but not in the liver (Figure 4). In epididymal adipose tissue, *Col24α1* was increased by 5-fold (Figure 4A), relative to a 1.5–2-fold increase in skeletal muscle including gastrocnemius (Figure 3A) and the superficial vastus lateralis (Figure 4C).

### 2.4. Col24α1 mRNA Was Increased in Visceral Adipose Tissue of Obese Diabetic Human Subjects

**Figure 5:**
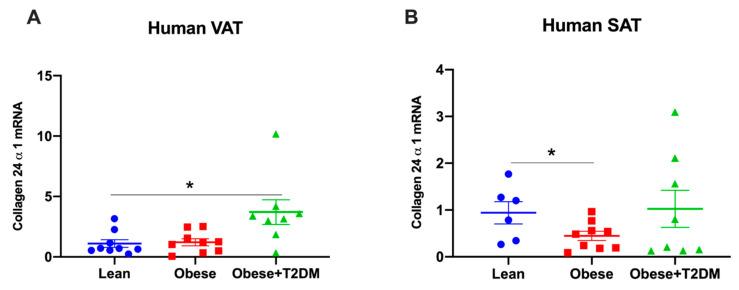
The mRNA levels of *Col24α1* in white adipose tissue of lean, obese, obese and diabetic subjects. *Col24α1* mRNA expression was measured in human visceral adipose tissue (VAT) ((**A**), *n* = 9:9:8) and subcutaneous adipose tissues (SAT) ((**B**), *n* = 6:9:8). Relative mRNA expressions were normalised to *18s*. Data were presented as means ± SEM, * = *p* < 0.05. VAT: visceral adipose tissue; SAT: subcutaneous adipose tissue; T2DM: Type 2 diabetes mellitus.

As *Col24α1* gene expression was most differentially upregulated in the adipose tissue of obese mice by HF diet, we assessed its expression in human adipose tissue (anthropometric data of human subjects shown in Supplemental Table S3). *Col24α1* mRNA was increased in the visceral adipose tissue, but not the subcutaneous adipose tissue of obese diabetic subjects compared to lean subjects (Figure 5). In contrast, *Col24α1* mRNA was decreased in the subcutaneous adipose tissue, but was unchanged in the visceral adipose tissue of obese subjects compared to lean subjects (Figure 5).

## 3. Discussion

Our current study applied an ECM-specific mass spectrometry-based proteomics to characterise the complete view of ECM protein changes associated with high fat (HF) diet feeding in mice. We identified, for the first time, significant differences in protein and pathway changes between muscle and liver. In the skeletal muscle, the ECM-related pathways were shown to be increased. Yet in the liver HF diet-associated changes were mainly pathways involved in metabolism. In particular, the ECM collagen changes were distinct between the insulin-resistant skeletal muscle and liver, where the majority of muscle collagen isoforms were increased whereas the liver collagens were differentially but moderately affected. Moreover, we identified a novel association between collagen 24α1 and insulin resistance in the skeletal muscle and adipose tissue. Increased collagen 24α1 was further seen in the visceral adipose tissue of obese diabetic subjects relative to lean controls, suggesting a potential role of collagen 24α1 in the pathogenesis of insulin resistance, obesity and Type 2 diabetes in mice and human.

Studies of the role of specific ECM components in insulin resistance are pivotal but incomplete. This is mainly due to limitations of current approaches that give a more global view of changes such as oligonucleotide microarray analysis and two-dimensional gel electrophoresis based tandem MS analysis (2-DE/MS-MS) [10,11], where oligonucleotide microarray analysis only detects changes in mRNA levels. Due to the low abundance of ECM proteins relative to most of the cytosolic proteins, detections of ECM proteins by 2-DE/MS-MS were difficult and insufficient. The key step in proteomic analysis of the ECM is their isolation. One traditional method is to homogenise the whole tissue then to separate the ECM by centrifugation [18]. The challenges with this method are contamination with proteins from damaged cells and the disruption of the ECM architecture. We refined methods to minimise contamination and disruption of the ECM using an in situ isolation approach by detergent, SDS [13], which leaves the ECM protein enriched and structure intact. Although preliminary due to the low sample size, our proteomics data are consistent with findings from other biochemical approaches specifically in skeletal muscle [19,20], therefore provide evidence for the ECM-based proteomics as a powerful tool in ECM-related research.

It is significant to see the different pathway changes between the insulin-resistant skeletal muscle and liver of obese mice. We detected less ECM proteins in muscle relative to liver, indeed fewer overall proteins (78 proteins in muscle relative to 265 proteins in liver, Supplemental Tables S1 and S2). This could be due to a more complex cellular components of the liver consisting of multiple cell types, primarily parenchymal cells (hepatocytes) and non-parenchymal cells (kupffer cells, hepatic stellate cells and liver sinusoidal endothelial cells). However, the skeletal muscle is primarily made of muscle fibres from myocytes surrounded by connective tissues. The majority of the ECM collagens were increased in muscle of obese mice suggesting a remodelling towards a more fibrotic state. This is consistent with various previous studies both in mice and human [19,21,22]. However, the liver of obese mice had a balanced and moderate remodelling which may result from more dynamic changes of cell types during obesity [23]. Indeed, the gene-annotation enrichment analysis suggests that the ECM-related pathways were only enriched in the muscle, but not in the liver, suggesting that the ECM remodelling may be more prominent and influential in the muscle. It is worth noting that various isoforms of keratin were also found to be increased in muscle of HF-fed mice by proteomics (Supplemental Table S1). Since no indication of a role of keratin in obesity and insulin resistance, future in-depth investigations will be important and are needed.

Increased collagen protein in insulin-resistant skeletal muscle was shown to be associated with increased mRNA levels. In gastrocnemius muscle, the mRNAs of *Col24α1*, *Col1α1* and *Col3α1* were increased by HF diet feeding. These results were consistent with previous results by us [4] and others [22]. The increased protein expression of Col1 and Col3 has also been shown in insulin-resistant skeletal muscle in human [22,24] and mice [4]. Increased collagen protein expression could also be due to decreased degradation by matrix metalloproteinases or MMPs. It has been shown that in skeletal muscle increased collagen protein was due to decreased enzymatic activity of MMP9 [4]. Moreover, genetic deletion of *mmp9* gene resulting in increased protein expression of Col3 and Col4 in the muscle, exacerbated HF diet-induced insulin resistance in muscle [25]. Taken together, these results suggest that our data are consistent with previous findings, yet identified a novel association between increased Col24α1 and muscle insulin resistance.

To further establish how Col24α1 may be associated with insulin resistance, we showed that Col24α1 interacts with Col1α1, Col1α2, Col2α1, Col3α1 and Col5α2 from a string networking analysis of the collagens [17]. These results suggest that Col24α1 may regulate or interact with other collagen isoforms that have been shown to be remodelled during muscle insulin resistance, therefore influencing metabolism during excess caloric intake such as HF feeding. Unfortunately, due to the unavailability of a functional collagen 24 antibody, we were unable to further investigate its regulation and interactome.

Although Col24α1 protein was increased by >10-fold in the muscle, its mRNA level was most increased in the white adipose tissue of mice (5-fold) when compared with gastrocnemius muscle (1.5-fold) and superficial vastus lateralis (2-fold). Increased *Col24α1* mRNAs in the white adipose tissue of obese mice were consistent with previously reported increases in mRNAs of other collagen isoforms including *Col5α2*, *Col6α2*, *Col6α3* and *Col18α1* [7,26,27,28]. While these studies identified crucial roles of collagens 6 and 18 in the pathogenesis of insulin resistance in obese mice, our studies imply a potential role of collagen 24 in diet-induced insulin resistance. Collagen 24 knockout mice will provide a valuable tool for future investigations.

It has been previously shown that increased collagen deposition is also manifested in human adipose tissue where *Col6α3* mRNA was increased in the subcutaneous abdominal and gluteal adipose tissue of metabolically obese subjects [29]. Here we showed that *Col24α1* mRNA was not changed in the visceral adipose tissue but decreased in the subcutaneous adipose tissue of obese subjects. These disparities could be due to differences in the study populations, where Khan et al. studied obese Asian Indians relative to Caucasian controls whereas our study compared obese subjects and lean controls of all Caucasian European descendants [29]. Interestingly, the mRNA levels of *Col24α1* were indeed increased in the visceral adipose tissue, but not in the subcutaneous adipose tissue of obese and diabetic subjects relative to lean controls. In humans, visceral adipose tissue is associated with insulin resistance, dyslipidaemia and Type 2 diabetes, while subcutaneous adipose tissue is associated with preserved insulin sensitivity and mitigates risk of metabolic disorders [30,31,32]. Moreover, in vitro study revealed that adipocytes from visceral adipose tissue rather than subcutaneous adipose tissue of obese mice showed impaired insulin-stimulated glucose uptake, adipogenesis ability and ECM remodelling representing dysfunctional adipocytes [33]. Thus, our result that *Col24α1* mRNA expression was increased in the visceral adipose tissue of obese and diabetic subjects but not in the subcutaneous adipose tissue implied a potential pathogenic role of Col24α1 in obesity and Type 2 diabetes. Col24α1 was previously identified as a minor type V collagen [34] and mice with genetic deletion of *Col24α1* were fertile, suggesting that it is not an essential structural protein of the ECM. Therefore, Col24α1 may represent a novel therapeutic target of obesity-related metabolic disorders.

In conclusion, using extracellular compartment-specific proteomics technique we characterised the complete view of ECM changes that were associated with HF diet feeding in skeletal muscle and liver of mice. The distinct signatures of collagen changes between muscle and liver highlight a different role of the ECM remodelling in different tissues of obesity. Furthermore, we identified a novel association between collagen 24α1 and insulin resistance in muscle and white adipose tissue, providing evidence for a potential role of collagen 24α1 in the pathogenesis of diet-induced insulin resistance in these tissues.

## 4. Materials and Methods

### 4.1. Animal Models

Male C57BL/6J mice were housed in a temperature (22 ± 1 °C) and humidity-controlled room with a 12 h light/dark cycle. Mice were fed either a chow control diet (D/811004; Special Diets Services, Essex, UK) containing around 13% total proteins, 84% carbohydrates and 3% fat or a 60% high fat (HF, primarily lard-based) diet (824054; Special Diets Services, Essex, UK) for 20 weeks starting at 6 weeks of age. Mice had free access to food and water. Only male mice were used in this study due to their high susceptibility to a robust insulin resistant phenotype after HF feeding. To confirm the hyperglycaemia phenotype, mice were fasted for 5 h in the morning before a blood glucose measurement from the tail vein. All procedures were performed under the UK Home Office Project Licence (P05F71E15; Date of issue 3 May 2019) following the ARRIVE guidelines and were approved by the Welfare and Ethical Use of Animals Committee at the University of Dundee.

### 4.2. ECM-Specific Mass Spectrometry-Based Proteomics

#### 4.2.1. Isolation of the ECM

Gastrocnemius muscle and liver were collected from 5 h fasted mice (morning fasting). Three or four mice were used for each dietary group. The ECM compartments of the tissues were isolated using an in situ approach by detergent SDS [13]. Gastrocnemius from one leg and ≈500 mg liver were incubated with 1% SDS at 4 °C for 5–7 days. The 1% SDS was changed daily. At the end of the incubation, tissue remains were weighed, frozen and grinded in liquid nitrogen. For proteomics studies, frozen samples were dissolved in 8 M guanidine with 100 mM dithiothreitol and heated. Iodoacetamide (200 mM) was then added and incubated for 45 min at room temperature in the dark. Samples were diluted to achieve a final concentration of 1M guanidine for trypsin digestion. In vivo quantitative proteomics of the ECM compartment in chow- and HF-fed mice were performed using iTRAQ (Isobaric tags for relative and absolute quantitation) labelling.

#### 4.2.2. iTRAQ Labelling Peptide Analysis by LC-MS/MS

Peptide concentration was determined by the bicinchoninic acid (BCA) assay and 100 μg peptides from each sample were used for the assay. Peptides were labelled with iTRAQ four-plex (AB Sciex, Warrington, UK) as previously described [35] and analysed by the LC-MS (Liquid chromatography-mass spectrometry) /MS [36]. Peptides were separated by an Easy nLC-1000 pump connected to an autosampler system (ThermoFisher Scientific, Bremen, Germany) and analysed by a Q-Exactive mass spectrometer (ThermoFisher Scientific, Bremen, Germany) equipped with nanoelectrospray. All resulting MS/MS spectra were assigned to peptides using the IPI (International Protein Index) mouse database version 3.83 by the PEAKS software v.8.5 (Bioinformatics solutions Inc, Waterloo, ON, Canada). Mass tolerance for precursor ions and fragment ions was set to 7 and 10 ppm, respectively. Searches of the MS/MS spectra incorporated fixed modifications of carbamidomethylation of cysteines and iTRAQ four-plex modification of lysines and peptide N termini, as well as the variable modifications of oxidation of methionine, and phosphorylation of serine, threonine and tyrosine residues. Samples (*n =* 3–4 mice per group) were run on two separate iTRAQ four-plex assays. All data were normalised to one sample from the chow-fed group that was run on both assays as inter-assay control for each protein. Changes of protein abundance caused by HF feeding was presented as the ratio of the mean of samples from HF-fed mice over the mean of samples from chow-fed mice.

### 4.3. Gene-Annotation Enrichment Analysis and KEGG Pathway Mapping

The Database for Annotation, Visualisation and Integrated Discovery (DAVID) v6.8 (https://david.ncifcrf.gov/home.jsp) was used for the pathway enrichment analysis [37,38]. Proteins that were shown with a ratio of HF/Chow > 2 from the proteomics study were converted to their corresponding gene IDs. The gene list was uploaded, and the functional annotation analysis was run using Mus musculus selected as the species and background. The KEGG pathway was mapped using count threshold of 3 and EASE (modified Fisher exact *p value*) threshold of 0.05.

### 4.4. String Network Analysis

Protein–protein associations were determined using the STRING (v.11.0) database (www.string-db.org; ELIXIR, Cambridgeshire, UK) [17], which collect, score and integrate all publicly available sources of protein–protein interaction information, and to complement these with computational predictions. The corresponding gene IDs were used as identifiers.

### 4.5. Mouse Tissue Collection and Preparation

Mouse tissues were collected from 5 h fasted mice (morning fasting). RNA was isolated from epididymal adipose tissue, gastrocnemius, superficial vastus lateralis or liver samples. Briefly, 30–100 mg tissues were weighed into 1 mL Trizol (93289; Sigma Aldrich, Dorset, UK), lysed by bullet blender and centrifuged at 13,000 rpm for 15 min. The lysates were then treated with chloroform, isopropanol and 75% ethanol to extract RNAs and the total RNAs were dissolved in 30 μL nuclease free water and concentration determined by nanodrop. Then, 1 μg total RNAs were transcribed to cDNAs by SuperScript TM Reverse Transciptase (18064022; ThermoFisher, Perth, UK), according to the manufacturer’s protocol. cDNAs were diluted 20 times before running real-time PCR.

### 4.6. Human Sample Collection and Preparation

Paired human subcutaneous and visceral adipose tissue samples were obtained from subjects undergoing elective abdominal surgery at the Royal Infirmary of Edinburgh. Superficial subcutaneous tissue was obtained at the incision between the overlying skin and the muscles of the anterior abdominal wall. Visceral tissue was obtained from either omental or mesenteric depot. Samples were obtained from the Edinburgh adipose tissue bank (part of the Lothian NRS Human Annotated Bioresource). Ethical approval was obtained from the East of Scotland Research Ethics Service (15/ES/0094, approved 15 July 2015) and informed consent was obtained from each participant. Samples were immediately frozen at −80 °C until analysis. Then, 30–50 mg tissues were used for total RNA extraction and 2 μg total RNAs were transcribed to cDNAs as described above.

### 4.7. Measurement of Gene Expression by Real-Time PCR 

Quantitative PCR were performed using the TaqMan^®^ Gene Expression assay (ThermoFisher, Perth, UK) for mouse collagen24α1 (Mm013237744_m1), human collagen24α1 (Hs00537706_m1), mouse collagen1α1 (Mm00801666_g1), mouse collagen1α2 (Mm00483888_m1), mouse collagen3α1 (Mm00802331_m1). 18s (Hs99999901_s1) was used as reference controls in human and mouse adipose tissue. Mouse GAPDH (Mm99999915_g1) was used as reference control in the other tissues. 

### 4.8. Statistical Analyses

Data were expressed as mean ± SEM. Proteomics data were presented as HF/Chow ratios. Real-time PCR data were calculated by 2^−ΔΔCt^ using 18 s or GAPDH as reference genes. Statistical analyses were performed using either unpaired Student’s *t*-test or one-way ANOVA as appropriate and figures were generated by GraphPad Prism software of v.8.4.2 (GraphPad Software, San Diego, CA, USA).The significance level was at *p* < 0.05.

## Figures and Tables

**Table 1:** Gene-annotation enrichment analysis and KEGG (Kyoto Encyclopaedia of Genes and Genomes) pathway mapping were performed on proteins that were increased by >2-fold in the skeletal muscle and liver of high fat (HF)-fed mice. The Database for Annotation, Visualisation and Integrated Discovery (DAVID) v6.8 was used for the analysis and modified Fisher exact *p value* < 0.05 was used as the threshold for significance. Count: numbers of genes involved in the term; %: % coverage of involved genes over total genes in the term; *p value*: modified Fisher exact *p value*, the smaller, the more enriched. Benjamini: FDR (false discovery rate) corrected *p value*s.

**Skeletal Muscle**						
**Category**	**Term**	**Count**	**%**	**Genes**	***p*** **Value**	**Benjamini**
KEGG_PATHWAY	Protein digestion and absorption	6	54.5	*COL3A1, COL1A2, COL2A1, COL1A1, COL24A1, COL5A2*	1.75 × 10^−10^	1.22 × 10^−9^
KEGG_PATHWAY	ECM–receptor interaction	6	54.5	*COL3A1, COL1A2, COL2A1, COL1A1, COL24A1, COL5A2*	1.75 × 10^−10^	1.22 × 10^−9^
KEGG_PATHWAY	Amoebiasis	6	54.5	*COL3A1, COL1A2, COL2A1, COL1A1, COL24A1, COL5A2*	7.48 × 10^−10^	2.62 × 10^−9^
KEGG_PATHWAY	Platelet activation	6	54.5	*COL3A1, COL1A2, COL2A1, COL1A1, COL24A1, COL5A2*	1.33 × 10^−9^	3.10 × 10^−9^
KEGG_PATHWAY	Focal adhesion	6	54.5	*COL3A1, COL1A2, COL2A1, COL1A1, COL24A1, COL5A2*	1.35 × 10^−8^	2.36 × 10^−8^
KEGG_PATHWAY	PI3K-Akt signalling pathway	6	54.5	*COL3A1, COL1A2, COL2A1, COL1A1, COL24A1, COL5A2*	1.93 × 10^−7^	2.70 × 10^−7^
**Liver**						
**Category**	**Term**	**Count**	**%**	**Genes**	***p*** **Value**	**Benjamini**
KEGG_PATHWAY	African trypanosomias-is	4	7.0	*HBA-A1, HBA-A2, APOA1, HBB-B2*	7.60 × 10^−4^	0.058
KEGG_PATHWAY	Biosynthesis of antibiotics	6	10.5	*LDHA, ALDOB, FBP1, ALDH2, HADH, ACAT1*	0.005	0.171
KEGG_PATHWAY	Glycolysis/Gluconeogenesis	4	7.0	*LDHA, ALDOB, FBP1, ALDH2*	0.005	0.118
KEGG_PATHWAY	Butanoate metabolism	3	5.3	*HADH, ACAT1, BDH1*	0.009	0.156
KEGG_PATHWAY	Metabolic pathways	14	24.6	*LDHA, SORD, GLUD1, ALDOB, FBP1, ACAT1, LAP3, ALDH1A1, PRDX6, BHMT, ALDH2, ATP5A1, HADH, BDH1*	0.010	0.141
KEGG_PATHWAY	Gap junction	4	7.0	*TUBB5, TUBA4A, TUBA1A, TUBA1C*	0.010	0.123
KEGG_PATHWAY	Fructose and mannose metabolism	3	5.3	*SORD, ALDOB, FBP1*	0.013	0.140
KEGG_PATHWAY	Pyruvate metabolism	3	5.3	*LDHA, ALDH2, ACAT1*	0.017	0.158
KEGG_PATHWAY	Carbon metabolism	4	7.0	*GLUD1, ALDOB, FBP1, ACAT1*	0.022	0.178
KEGG_PATHWAY	Tryptophan metabolism	3	5.3	*ALDH2, HADH, ACAT1*	0.025	0.179
KEGG_PATHWAY	Malaria	3	5.3	*HBA-A1, HBA-A2, HBB-B2*	0.026	0.170
KEGG_PATHWAY	Fatty acid degradation	3	5.3	*ALDH2, HADH, ACAT1*	0.027	0.163
KEGG_PATHWAY	Lysine degradation	3	5.3	*ALDH2, HADH, ACAT1*	0.030	0.167
KEGG_PATHWAY	Valine, leucine and isoleucine degradation	3	5.3	*ALDH2, HADH, ACAT1*	0.033	0.172

## Supplementary Materials

Supplementary Materials can be found at https://www.mdpi.com/1422-0067/21/16/5738/s1.

## Abbreviations

Akt	Protein kinase B
BCA	Bicinchoninic acid
ECM	Extracellular matrix
HF	High fat
iTRAQ	Isobaric tags for relative and absolute quantitation
KEGG	Kyoto Encyclopaedia of Genes and Genomes
LC-MS	Liquid chromatography-mass spectrometry
MMPs	Matrix metalloproteinases
MMP9	Matrix metalloproteinases 9
MSPI3K	Mass spectrometry
PI3K	Phosphoinositide 3-kinase
SAT	Subcutaneous adipose tissue
SDS	Sodium dodecyl sulfate
2-DE	Two-dimensional gel electrophoresis
VAT	Visceral adipose tissue
WAT	White adipose tissue
